# Naso- and oropharyngeal bacterial carriage in nursing home residents: Impact of multimorbidity and functional impairment

**DOI:** 10.1371/journal.pone.0190716

**Published:** 2018-01-05

**Authors:** Anja Kwetkat, Wolfgang Pfister, Diana Pansow, Mathias W. Pletz, Cornel C. Sieber, Heike Hoyer

**Affiliations:** 1 Department of Geriatric Medicine, Jena University Hospital, Jena, Thuringia, Germany; 2 Institute of Medical Microbiology, Jena University Hospital, Jena, Thuringia, Germany; 3 Centre for Infectious Diseases and Infection’s Control, Jena University Hospital, Jena, Thuringia, Germany; 4 Institute of Biomedicine of Ageing, Friedrich-Alexander University Erlangen-Nuremberg, Nuremberg, Bavaria, Germany; 5 Institute of Medical Statistics, Computer Sciences and Documentation, Jena University Hospital, Jena, Thuringia, Germany; Leibniz-Institute DSMZ, GERMANY

## Abstract

**Objective:**

From April 2013 to February 2014 we performed a multicentre prospective cross-sectional study in 541 German nursing home residents. We determined pharyngeal carriage of *Streptococcus pneumoniae* (primary objective) and other bacteria (secondary objective) in naso- and oropharyngeal swabs by culture-based standard procedures and explored the influence of multimorbidity and functional status on bacterial carriage.

**Methods:**

Socio-demographic data, vaccination status, multimorbidity, nutrition and functional status defined by Comprehensive Geriatric Assessment were evaluated. We estimated carriage rates with 95% confidence intervals (CI) and explored potential risk factors by logistic regression analysis.

**Results:**

Pneumococcal post-serotyping carriage rate was 0.8% (95%CI 0.2–1.9%; 4/526). Serotyping revealed serotypes 4, 7F, 23B and 23F and *S*. *pseudopneumoniae* in two other cases. Odds of carriage were higher in men (Odds ratio OR 5.3 (95%CI 0.9–29.4)), in malnourished residents (OR 4.6 (0.8–25.7)), residents living in shared rooms (OR 3.0 (0.5–16.5)) or having contact with schoolchildren (OR 2.0 (0.2–17.6)). The most frequent pathogen was *Staphylococcus aureus* (prevalence 29.5% (25.6–33.6%)) with meticillin-resistant *Staphylococcus aureus* prevalence of 1.1%. Gram-negative bacteria (GNB) were found in 22.5% (19.0–26.3%) with a prevalence of extended-spectrum beta lactamase (ESBL) producing bacteria of 0.8%. Odds of *S*. *aureus* carriage were higher for immobility (OR 1.84 (1.15–2.93)) and cognitive impairment (OR 1.54 (0.98–2.40)). Odds of GNB carriage were higher in residents with more severe comorbidity (OR 1.13 (1.00–1.28)) and malnutrition (OR 1.54 (0.81–2.91)).

**Conclusions:**

Given the observed data, at least long-term carriage of *S*. *pneumoniae* in nursing home residents seems to be rare and rather unlikely to cause nursing home acquired pneumonia. The low rate of colonization with multi drug resistant (MDR) bacteria confirms that nursing home residency is not a risk factor for MDR pneumonia in Germany. For individual risk assessment in this susceptible population, immobility and malnutrition should be considered as signs of functional impairment as well as comorbidity.

## Introduction

Community acquired pneumonia (CAP) in older persons increases with advanced aging [1] in industrial countries. CAP is a life threatening infection associated with a mortality of up to 30% in old age [2]. Nursing home residents suffering from nursing home acquired pneumonia (NHAP) represent a subgroup of particular interest: NHAP incidence is approximately 1/1000 resident days [3, 4], and mortality ranges from 44% in Germany [2] to 55% in the USA [5]. Patients with NHAP suffer from more comorbidities, more severe functional impairment and often have a more severe clinical course [2, 3, 6]. Although airborne droplet infection is common in younger individuals, pneumonia in old persons is often caused by aspiration of colonized oropharyngeal contents [7]. Dysphagia and aspiration are common disorders in nursing home residents and can explain the high incidence of NHAP [8]. Evaluating oropharyngeal bacterial carriage among nursing home residents may thus be useful to identify potential pathogens of NHAP. This could be helpful to initiate appropriate antibiotic treatment.

There is an ongoing debate, if nursing home residency itself is a risk factor for pneumonia with multidrug resistant (MDR) pathogens. Data from the United States indicate an excess of MDR pathogens and Gram-negative bacteria (GNB) in NHAP [6], but European studies do not confirm these findings [2, 6, 9]. Overall, *Streptococcus pneumoniae* is the most frequent causative pathogen in CAP and NHAP worldwide [6].

Pneumococcal carriage and pneumococcal serotype distribution are of particular interest as two types of pneumococcal vaccines for adults are available with different serotype coverage: 23-valent pneumococcal polysaccharide vaccine (PPV23) vs. 13-valent pneumococcal conjugate vaccine (PCV13). The knowledge of pneumococcal serotype distribution is relevant to estimate the potential benefit of broader serotype coverage of vaccine. As vaccination of children with pneumococcal conjugate vaccine leads to indirect herd protection of adults [10], in some countries pneumococcal vaccination is recommended for children only, e.g. in the Netherlands. In Germany, the Standing Committee on Vaccination (STIKO) generally recommended pneumococcal conjugate vaccine for children since 2006. The actual vaccination coverage rate is about 70% in children [11]. In 2013/2014, the period of our study, the STIKO recommended the 23-valent pneumococcal polysaccharide vaccine (PPV23) for seniors 60 years of age and above as routine immunization. The German Health Interview and Examination Survey for Adults (DEGS1) showed a pneumococcal vaccination rate of 31.4% among the group of the 65 to 79 years old Germans [12].

Data suggest that infants are the source of pneumococcal transmission to adults [13]. To the authors’ knowledge, there are no data concerning transmission to nursing home residents. We supposed that nursing home residents with multimorbidity and functional impairment have rare contact with infants because of their limited mobility and range of motion. Thus, we hypothesized that high levels of multimorbidity and a low functional status among nursing home residents influence bacterial naso- and oropharyngeal carriage.

Our observational cross-sectional study primarily assessed the prevalence of nasopharyngeal or oropharyngeal carriage with *S*. *pneumoniae* and its serotype distribution in nursing home residents. As a secondary objective, we determined nasopharyngeal or oropharyngeal carriage with other bacteria and investigated the effects of multimorbidity and functional status on bacterial carriage.

## Methods

### Study design, setting and participants

Written informed consent by the participants or the legal guardian and local ethics committee approval were obtained (3659-01/13, Ethikkommission der Friedrich-Schiller-Universität Jena an der Medizinischen Fakultät). The study was registered at the German Clinical Trials Register (DRKS00004833).

From April 2013 until February 2014, we performed a prospective, cross-sectional, multi-centre, observational study in Thuringia, Germany. Postal invitation was sent to 46 regional nursing homes, of which 22 finally participated. The primary outcome was the total and serotype-specific nasopharyngeal or oropharyngeal *S*. *pneumoniae* carriage rate in nursing home residents. The spectrum of other pathogens (secondary outcome) was detected by culture-based methods. Furthermore, the influence of multimorbidity and functional status of residents as potential risk factors for pathogen carriage was explored. Participants were nursing home residents 65 years of age and above without exclusion criteria (i.e. lack of power of judgment without legal guardian, refusal of participation by legal guardian, use of systemic immunosuppressant drugs, antibiotic use up to ten days preceding nasopharyngeal swab, hospital stay up to two weeks preceding nasopharyngeal swab and a diagnosis of terminal illness).

### Assessment

A skilled study nurse and a physician reviewed the medical charts of all participants for socio-demographic data, vaccination status for pneumococcal and influenza vaccination, antibiotic use, and for evaluation of multimorbidity by Cumulative Illness Rating Scale for geriatric patients (CIRS-G) [14]. They obtained naso- and oropharyngeal swabs and performed the comprehensive geriatric assessment (i.e. activities of daily living Barthel Index [15], Timed up & go Test [16], Mini Mental State Examination [17], and the Mini Nutritional Assessment Short Form (MNA-SF) [18].

### Nasopharyngeal and oropharyngeal swabs and laboratory analysis

Oropharyngeal swabs were obtained using sterile tongue blades and sterile cotton swabs (Amies transport swabs; Nerbe plus, Winsen/Luhe, Germany). Nasopharyngeal swabs were obtained in accordance with WHO standard method [19]. After sampling, swabs were immediately inoculated in transport medium, stored at room temperature and brought within 12 hours to the Institute of Clinical Microbiology, Jena University Hospital. The specimens were streaked out and cultured on different agar plates. Columbia blood agar (Oxoid, Wesel, Germany) was used for detection of *Streptococcus*, *S*. *pneumoniae*, *Moraxella* and others, boiled-blood-agar for the presence of *Haemophilus* strains; MacConkey-Agar (Oxoid) for presence of gram-negative bacteria (GNB); MRSA selective agar (Oxoid) for presence of meticillin resistant *Staphylococcus aureus* (MRSA), and on VRE selective agar (Enterococcosel agar; BectonDickinson, Heidelberg, Germany) for presence of vancomycin resistant enterococci (VRE). Colonies were identified after 24 and 48 hours, respectively, using routine laboratory methods and an automatic analyser Vitek 2, bioMerieux Deutschland GmbH, Germany. In few swabs single bacteria species showed such a heavy growth that analysis of other species was not possible. In that case bacterial carriage of affected species was defined as missing data. Evaluation of antimicrobial susceptibility of all samples was based on broth microdilution series to determine minimal inhibitory concentration using Vitek 2 and E-Test, respectively. After identification of isolates, *Streptococcus pneumoniae*, *Staphylococcus aureus*, enterococci, Enterobacteriaceae and *Pseudomonas aeruginosa* were tested for particular antibiotic resistance (penicillin and macrolide resistance of pneumococci; meticillin resistant *Staphylococcus aureus* (MRSA); vancomycin resistant enterococci (VRE); extended-spectrum beta-lactamase producing (ESBL) Enterobacteriaceae; carbapenem resistance of *Pseudomonas aeruginosa*). Pneumococci were tested for their antimicrobial susceptibility to penicillin G, cefotaxime, clindamycin, erythromycin, levofloxacin, linezolid, tetracycline, and sulfamethoxazole-trimethoprim. All other bacterial strains were tested for their antimicrobial susceptibility by using the routinely applied antibiotic panel at the Jena University Hospital. Resistance testing was conducted by VITEK 2 using the species specific panels applied from the manufacturer bioMerieux, Marcy l`Etoile, France. Pneumococcal serotyping was performed by the National Reference Centre for Streptococci, Aachen, Germany. The pneumococci isolates were cultured on sheep blood agar and were re-identified by optochin susceptibility and bile solubility. If there was doubt in identification, 16srDNA- and sodA-sequencing were performed. Serotyping was obtained by Neufeld’s Quellung reaction using type and factor sera provided by the Statens Serum Institute, Copenhagen, Denmark. Serotyping by multiplex PCR was performed in accordance with www.cdc.gov/ncidod/biotech/strep/pcr.htm.

### Statistical analysis

Expecting a pneumococcal prevalence of 10% to 20% [13, 20, 21] a sample size of 500 subjects allowed us to calculate a two-sided 95% confidence interval (CI) extending the observed prevalence by +/- 2.6 to 3.5%. Mean and standard deviation, median and interquartile range, and frequencies were used where appropriate. The prevalence of carriage was estimated as the proportion of participants with microbiologic evidence of pathogens from nasopharyngeal or oropharyngeal swabs out of all participants with valid culture-based results. Exact 95% CI was calculated assuming a binomial distribution. For the analysis of associations the total score of CIRS-G (0 to 56) and the total number of severely affected organ systems rated 3 or 4 in CIRS-G (0 to 14) were used to quantify multimorbidity. The functional status was operationalized by the following variables: Requirement of high level care (Barthel-Index 0–30 vs. >30–100), immobility (Timed up & go Test not feasible vs. feasible) and malnutrition (MNA-SF<8 vs. ≥8). Logistic regression models were used to analyse the association between pathogen carriage and potential influencing factors. The strength of association was estimated by the odds ratio (OR). According to the explorative approach 95% CI were reported instead of p-values. Due to the low number of events only crude ORs for single factor regression were calculated for the pneumococci carriage. In case of zero cell counts 0.5 was added to each cell. Multiple logistic models could be fitted for *S*. *aureus*, gram negative bacteria (GNB) and *E*.*coli* owing to their higher prevalence. A predefined set of covariates was considered for modelling. We report the final models resulting from backward selection excluding covariates with p≥0.2.

## Results

### Participants

We enrolled 541 participants out of 2,131 residents in 22 participating Thuringian nursing homes which accommodated between 42 to 275 residents. The participants met all inclusion criteria and no exclusion criteria. Their mean age was 84.5 years (range 65–103 years), 27.9% were males. Further characteristics are summarized in Table 1. About 50% of residents suffered from three or more severe medical problems. The most affected systems were musculoskeletal/skin (91.3%), vascular (90.2%), psychiatric illness (79.7%), genitourinary (71.3%) and neurological (50.5%). Oropharyngeal and nasopharyngeal swabs were taken from 539 (99.6%) and 529 (97.8%) participants, respectively.

**Table 1:** Characteristics of participants.

Variables	Study population (N = 541)
Age [y], mean (SD)	84.5 (7.5)
Male gender, n (%)	151 (27.9)
Duration of stay in nursing home y, (N = 540), mean (SD)	3.3 (4.4)
Level of care, n (%) No I II III	- 5 (0.9) 246 (45.5) 214 (39.6) 76 (14.0)
Marital status, n (%) Single Married Widowed Divorced	- 69 (12.8) 98 (18.1) 329 (60.8) 45 (8.3)
Living in shared rooms, n (%)	221 (40.9)
Stomata, n (%) Tracheostomy PEG-/ nasal probe Enterostomy	- 2 (0.4) 25 (4.6) 5 (0.9)
Vaccinated against, n (%)^a^ Influenza (current season) Pneumococci (PPV23)	- 272 (50.3) 103 (19.0)
Antibiotic use 3 months up to 10 days preceding swabs, n (%)	81 (15.0)
Contact to children at least once a month Infants Schoolchildren	- 39 (7.2) 48 (8.9)
Body mass index <19 kg/m^2^, n (%)	28 (5.2)
Malnutrition according to MNA-SF<8, n (%)	59 (10.9)
Multimorbidity by CIRS-G sum score, mean (SD)	17.7 (5.7)
Number of CIRS-G risk categories 3 or 4, median (IQR)	3 (2–4)
Cognitive state by MMSE sum score (N = 517), median (IQR)	20 (9–27)
Cognitive impaired residents (MMSE<24) (N = 517), n (%)	310 (60)
Immobile residents (timed up and go test not feasible), n (%)	258 (47.7)
Barthel-Index, median (IQR)	55 (20–75)
Requiring high-level care (Barthel-Index 0–30), n (%)	181 (33.5)

CIRS-G—Cumulative Illness Rating Scale for Geriatrics; IQR—interquartile range;

PPV23–23-valent pneumococcal polysaccharide vaccine; MNA-SF—Mini Nutritional Assessment Short Form; MMSE—Mini Mental State Examination; PEG—percutaneous endoscopic gastrostomy; SD—standard deviation;

^a^ Unknown for influenza and pneumococcal vaccination in 108 and 374 residents, respectively; percentage of vaccinated is based on N = 541

### Bacterial carriage

Table 2 summarizes frequency and prevalence of pathogen carriage. N positive is the number of participants with evidence of the specific pathogen in at least one—the nasopharyngeal and/or the oropharyngeal—swab. N valid is the number of subjects with well-defined results of laboratory analysis. This is the sum of N positive and the number of participants with both swabs available and clearly assessed as negative. According to this rule N valid varies slightly between 523 and 529 for several pathogens.

**Table 2:** Spectrum of pathogens in nasopharyngeal or oropharyngeal swabs isolated by culture-based standard procedures.

Pathogen	N valid	N positive	Prevalence [%]	95% CI [%]
**Gram positive**				
*Streptococcus pneumoniae*	526	6	1.1	0.4–2.5
*Staphylococcus aureus*	529	156	29.5	25.6–33.6
MSSA	529	150	28.4	24.5–32.4
MRSA	529	6	1.1	0.4–2.5
ß-haemolytic streptococci	525	17	3.2	1.9–5.1
*Bacillus* spp.	526	4	0.8	0.2–1.9
B-streptococci	526	5	1.0	0.3–2.2
**Gram negative**				
Any gram negative	525	118	22.5	19.0–26.3
Enterobacteriae				
*Escherichia coli*	524	43	8.2	6.0–10.9
Without ESBL	524	40	7.6	5.5–10.3
With ESBL	524	3	0.6	0.1–1.7
*Proteus mirabilis*	524	22	4.2	2.6–6.3
*Klebsiella peumoniae*	524	14	2.7	1.5–4.4
*Klebsiella oxytoca*	524	8	1.5	0.7–3.0
Without ESBL	524	7	1.3	0.5–2.7
With ESBL	524	1	0.2	0.0–1.1
*Enterobacter amnigenus*	524	3	0.6	0.1–1.7
*Enterobacter cloacae*	523	13	2.5	1.3–4.2
*Enterobacter aerogenes*	524	4	0.8	0.2–1.9
*Serratia marcescens*	526	2	0.4	0.0–1.4
*Citrobacter koseri*	526	3	0.6	0.1–1.7
*Serratia liquefaciens*	526	2	0.4	0.0–1.4
*Providencia stuartii*	526	1	0.2	0.0–1.1
*Morganella morganii*	526	3	0.6	0.1–1.7
Without ESBL	526	2	0.4	0.0–1.4
With ESBL	526	1	0.2	0.0–1.1
*Raoultella planticola*	526	1	0.2	0.0–1.1
*Hafnia alvei*	526	1	0.2	0.0–1.1
*Pantoea agglomerans*	526	1	0.2	0.0–1.1
*Providencia rettgeri*	526	1	0.2	0.0–1.1
**Non fermenters**				
*Acinetobacter* spp.	525	10	1.9	0.9–3.5
*Pseudomonas aeruginosa*	524	16	3.1	1.8–4.9
*Pseudomonas stutzeri*	526	1	0.2	0.0–1.1
*Stenotrophomonas maltophilia*	526	2	0.4	0.0–1.4
**Fungi**				
*Candida* spp.	527	6	1.1	0.4–2.5

N valid—Number of participants with well-defined results from laboratory analysis; N positive—Number of participants with evidence of pathogen; MSSA—meticillin sensitive S. aureus, MRSA—meticillin resistant S. aureus, ESBL—extended-spectrum β-lactamase, CI—confidence interval

*S*. *pneumoniae* was isolated by culture in nasopharyngeal (6) and oropharyngeal (1) swabs of six out of 526 residents resulting in a prevalence of 1.1% (95%CI 0.4–2.5%). No antibiotic resistance was found. Subsequent serotyping revealed *S*. *pseudopneumoniae* in two cases and serotypes 4, 7F, 23B and 23F otherwise (post-serotyping prevalence 0.8%, 95%CI 0.2–1.9%). Residents most frequently carried *S*. *aureus* (29.5%, 95%CI 25.6–33.6%) followed by *E*. *coli* (8.2%, 95%CI 6.0–10.9%). MRSA was identified in two oropharyngeal and four nasopharyngeal swabs of six residents (1.1% (0.4–2.5%)). ESBL producing gram negative pathogens were found in four residents (3 *E*. *coli*, 1 *Klebsiella oxytocae* and *Morganella morganii*). The normal upper respiratory tract flora comprised *S*. *viridans* (98%), coagulase negative staphylococci (95.5%), neisseriae (64.3%), enterococci (52.7%), corynebacteria (46.6%), *Haemophilus parainfluenzae* (12.3%) and stomatococci (6.5%).

### Associations between resident-related factors and pathogen carriage

#### Carriage with *S*. *pneumonia*

The odds of pneumococcal carriage (= risk of carriage/(1-risk of carriage)) were higher in men (4/142) vs. women (2/378), in residents with a body mass index < 19 kg/m^2^ (1/24) vs. higher (5/496), living in shared (4/208) vs. single room (2/312) or having contact with schoolchildren at least once a month (1/47) vs. less (5/473). Longer stay in nursing home residence (mean of 2 years in carriers vs. 3.2 years in non-carriers) and vaccination against pneumococci (0/99) vs. none (2/61) were associated with lower odds for carriage. No association with pneumococcal carriage was observed for age, antibiotic use preceding swabs, influenza vaccination and contact with infants. Multimorbidity was higher in non-carriers than in carriers (mean CIRS-G 17.7 vs. 14.9, mean number of severely affected organ systems 3 vs. 2.5). Malnutrition (MNA-SF <8 points), immobility (timed up and go test not feasible) and the requirement of high level of care (Barthel-index 0–30) were identified as indicators of low functional status. Higher odds of carriage were found in residents with malnutrition (2/51) vs. no malnutrition (4/469). Immobility and high level of care requirement were not associated with pneumococcal carriage. Table 3 summarizes the odds ratios from univariate logistic regression analysis which quantify the strength of the association.

**Table 3:** Associations between resident-related factors and pathogen carriage (explorative logistic regression models).

Predefined set of covariates	*Streptococcus pneumoniae* (culture)	*Staphylococcus aureus*	Any gram negative	*Escherichia coli*
	(Carriers: 6 of 526) Crude OR (95% CI)	(Carriers: 150 of 506) Adjusted^a^ OR (95% CI)	(Carriers: 118 of 524) Adjusted^a^ OR (95% CI)	(Carriers: 43 of 523) Adjusted^a^ OR (95% CI)
Age per year	0.99 (0.88–1.10)	0.98 (0.95–1.01)	ex	ex
Male gender	5.32 (0.96–29.4)	ex	ex	2.05 (1.07–3.91)
Duration of stay in nursing home per year	0.85 (0.54–1.34)	0.95 (0.89–1.01)	ex	ex
Living in shared rooms	3.00 (0.54–16.6)	0.70 (0.46–1.06)	ex	ex
Antibiotic use preceding swabs^b^	1.13 (0.13–9.81)	ex	ex	ex
Influenza vaccination		ex	ex	ex
• yes vs. no	0.22–5.04)			
• unknown vs. no	0.30 (0.01–6.23)			
Pneumococcal vaccination (PPV23)		ex	ex	ex
• yes vs. no	0.00–2.62)			
• unknown vs. no	0.31 (0.06–1.48)			
Contact with infants at least once a month	0.94 (0.05–17.0)	ex	ex	ex
Contact with schoolchildren at least once a month	2.01 (0.23–17.6)	ex	ex	ex
CIRS-G: Per point of sum score	0.91 (0.78–1.07)	ex	ex	ex
CIRS-G: Per additional severely affected organ system^c^	0.84 (0.49–1.41)	0.85 (0.73–0.99)	1.13 (1.00–1.28)	ex
Requiring high-level care (Barthel-Index 0–30)	1.05 (0.19–5.78)	ex	ex	ex
Immobility^d^	1.13 (0.22–5.66)	1.84 (1.15–2.93)	ex	1.73 (0.90–3.29)
Body mass index<19 kg/m^2^	4.13 (0.46–36.8)	ex	ex	2.82 (0.98–8.07)
Malnutrition (MNA-SF <8)	4.60 (0.82–25.8)	ex	1.54 (0.81–2.91)	ex
Cognitive impairment (MMSE<24)	-	1.54 (0.98–2.40)	-	-

OR—odds ratio; CI—confidence interval; PPV23–23-valent pneumococcal polysaccharide vaccine; CIRS-G—Cumulative Illness Rating Scale for Geriatrics; MNA-SF—Mini Nutritional Assessment Short Form; MMSE—Mini Mental State Examination; ex—excluded by backward selection;

^a^ Adjusted odds ratios for variables remained in the multiple model after backward selection excluding those with p≥0.2

^b^ Within 3 months up to 10 days before swab

^c^ Total number of severely affected organ systems rated 3 or 4 in CIRS-G

^d^ Timed up and go test not feasible

#### Carriage with *S*. *aureus*

Higher odds for *S*. *aureus* carriage were found for immobility (OR 1.84) and cognitive impairment (OR 1.54), whereas higher age (OR 0.98 per year), longer stay in nursing home (OR 0.95 per year), living in shared rooms (OR 0.7) and higher multimorbidity (OR 0.85 per additionally severely affected organ system) were associated with lower odds for *S*. *aureus* carriage. Due to the low MRSA prevalence no further analysis was done.

#### Carriage with Gram-negative bacteria

Residents with more severely affected organ systems (OR 1.13) and malnutrition (OR 1.54) more likely carried at least one GNB. Owing to the small number of residents with stomata this potential risk factor was not considered for modelling. However, it is worth mentioning that both residents with tracheostoma, 13 of 25 with PEG-/nasal probe and 3 of 5 with enterostoma carried GNB.

#### Carriage with *E*. *coli*

*E*.*coli* was the second frequent residential pathogen and the most frequent Gram negative germ with clinical relevance. Thus, *E*.*coli* was included in the association analysis. According to the explorative logistic regression models male gender (OR 2.05), BMI<19 kg/m^2^ (OR 2.82) and immobility (OR 1.73) were associated with higher risk of *E*.*coli* carriage.

## Discussion

With a post-serotyping prevalence of 0.8%, we found a quite lower pneumococcal carriage rate than expected [13, 20, 21]. However, our study confirmed the results of recent studies in seniors [22–24], nursing home residents [23] and geriatric patients [25] revealing carriage rates between 0% and 2.3%. To improve detection of pneumococcal carriage, we used nasopharyngeal and oropharyngeal swabs as previously suggested [26] taken by a specifically trained study nurse [19]. Thus, a relevant systematic bias in estimation of the pneumococcal prevalence seems less probable. In concordance with Almeida et al. [23], we also found a diversity of serotypes. Neither of the currently available vaccines covers serotype 23B, that actually gains importance in invasive pneumococcal infections in adults [27, 28]. All other detected pneumococcal serotypes are covered by the PPV23 vaccine as well as the PCV13 vaccine. The pathogenic significance of *S*. *pseudopneumoniae* in respiratory tract diseases is still unclear [29]. According to these scarce data, we cannot substantially contribute to the discussion of the favourable vaccine in elderly nursing home residents. Given the observed data, at least long-term carriage of *S*. *pneumoniae* in nursing home residents seems to be rare and rather unlikely to cause nursing home acquired pneumonia. It could be suspected that temporary carriage introduced by visitors or the nursing home staff may be possibly responsible.

Our study did not reveal a significant relationship between pneumococcal carriage and functional status in the nursing home residents because of the low prevalence rate. In a recent study in older probably less impaired adults living independently in retirement communities the pneumococcal carriage rate was 1.9% [24], about twice as high compared to our study, but even low altogether. Our results might support the impression that residents with less severe comorbidity appear to be more frequently pneumococcal carriers. We also analysed the contacts with children. Unvaccinated they are known to have the highest carriage rates and may be the source for pneumococcal transmission to adults [13]. On the other hand, contact with vaccinated children seems to be relevant for the indirect herd protection of unvaccinated adults [10]. We found an OR of 2.0 (95% CI 0.23–17.6) for pneumococcal carriage of residents having contact with school children at least once a month. However, the number of pneumococcal carriers as well as the number of contacts with children seemed to be too small to provide evidence for the transmission or herd protection hypotheses.

The most frequent colonizing pathogen in our study was *S*. *aureus* with 28.4% MSSA colonization and 1.1% MRSA colonization. Our results are in accordance with a MSSA prevalence of 36.6% published in another German study [30]. In contrast, a UK study showed a prevalence of 16.2% [31]. So generally, attention should be paid to this high level of *S*. *aureus* carriage as it can be considered as a large reservoir of potential multiresistant pathogens [32] in naso- and oropharyngeal carriage of nursing home residents. The MRSA prevalence of 1.1% in our study is in line with several German studies [30, 33–35]. Thus, MRSA prevalence in Germany is much lower than that in the USA (27.5%[36]) or the UK (19% to 22%[37]). Our results may underestimate the MRSA prevalence by limiting the swabs to nose and oropharynx. Yet, it can be concluded that the prevalence in our study will be less than in USA as 65% to 85% of those colonized at other sites were also nasal carriers [36].

Only few studies evaluated age, functional status and multimorbidity as potential risk factors for *S*. *aureus* carriage and their results are mostly contradicting. One study reported a higher MSSA prevalence with higher age [31], the other one showed no influence [30]. The latter results were in accordance with our findings. Similarly controversial is the influence of cognitive impairment. We showed a higher risk for *S*. *aureus* carriage in residents with cognitive impairment (OR 1.54). In contrast, Lasseter et al. [31] did not find an association of cognitive deficits measured by MMS-short test. Daeschlein et al. [30] reported even a protective effect of vascular cognitive impairment. Probably, these discrepancies may be due to differences in evaluation of cognitive function. Immobility seems to be a risk factor for *S*. *aureus* carriage in our study supported by results of another German study [30]. Increased multimorbidity, a factor not evaluated in other studies, appeared to be protective (OR 0.85 per additionally severely affected organ system). That could be interpreted as contradictory because it may be expected that multimorbidity and immobility would have a similar effect. El-Solh et al. [38] showed, that pneumonia caused by *S*. *aureus* and GNB tended to be more frequently present as the functional status worsened. Therefore, the influence of functional aspects on *S*. *aureus* carriage was probably underestimated in the past and might be more relevant than multimorbidity.

We detected a GNB prevalence of 22.5% with *E*.*coli* being the most frequently isolated pathogen with a prevalence of 8.2%. Multiresistant GNB were exclusively due to ESBL with a prevalence of 0.8%. Therefore, they seem to play only a marginal role in naso- and oropharyngeal carriage of German nursing home residents. However, there are only a few data on oropharyngeal GNB carriage among nursing home residents. O’Donoghue et al.[39] reported an oropharyngeal GNB prevalence of 36.7% in Irish nursing home residents with *E*.*coli* as leading pathogen and polypharmacy (> 8 medications) and a history of ischemic heart disease as risk factors. According to our results, malnutrition (OR 1.5), more severely affected organ systems (OR 1.13) and indwelling devices appeared as risk factors for GNB carriage, whereas *E*.*coli* carriage was associated with a reduced BMI (OR2.8), male sex (OR 2.1) and immobility (OR 1.73). Interpreting polypharmacy as a surrogate marker for more severe illness we see the results of O’Donoghue et al. [39] in line with ours. To our knowledge, there are no further studies dealing with the influence of functional status or multimorbidity on pharyngeal GNB carriage. However, there are several studies concerning the prevalence of GNB as pathogen of CAP [40] and NHAP [2], but without evaluation of functional issues. Only El-Solh et al. [38] demonstrated the increase of *S*. *aureus* and GNB prevalence as pathogen of pneumonia in patients with worsened functional status. Considering the high carriage rate of *S*. *aureus*, GNB and *E*.*coli* and the possible influence of functional impairment and multimorbidity on carriage, the relevance of *S*. *aureus* and GNB as pathogen of CAP and NHAP should be re-evaluated. We think that published studies of pathogens causing community or nursing home acquired pneumonia might suffer from selection bias for the following reasons. Nursing home residents were often excluded as they are mainly not eligible for a chest X-ray in an erect postero-anterior and lateral projection which is the gold standard for diagnosing pneumonia [41]. Our data demonstrated that the majority of nursing home residents suffered from impaired mobility. Most of them would be ineligible for this diagnostic procedure. Particularly these residents have an increased risk of *S*. *aureus* and *E*.*coli* carriage. Furthermore, the spectrum of pneumonia causing pathogens may be biased due to missing sputum samples. Different studies showed that patients with pneumonia but without respiratory samples are significantly older, are more frequently nursing home residents, and suffer from more comorbidities [2, 40].

There are several limitations of our study. The cross-sectional observational design did not allow drawing causal inferences. The unexpectedly low pneumococcal carriage rate limits the analysis of multimorbidity and functional status as independent risk factors. However, the prospective approach, the sample size as well as the high participation rates strengthen this study. To reduce bias, the study team was well trained to take the swabs and apply the standardized comprehensive geriatric assessment. They were unaware of any microbiological results during the examination.

## Conclusion

This study confirms recent findings of low pneumococcal carriage rate in nursing home residents. Given the observed data, at least long-term carriage of *S*. *pneumoniae* in nursing home residents seems to be rare and rather unlikely to cause nursing home acquired pneumonia.

Furthermore, we found a low carriage rate of MRSA and ESBL producing Gram negative bacteria, despite the carriage rates of MSSA and GNB were high. This confirms that in Germany, nursing home residency is not a risk factor for MDR colonization and possibly resulting nursing home acquired pneumonia with multidrug resistant pathogens. The carriage of *S*. *aureus* and GNB was probably associated with multimorbidity and immobility. Therefore, our data provides support that multimorbidity and functional status may be crucial issues for carriage with *S*. *aureus* and GNB as potential pneumonia pathogens and probably more relevant than the place of residency.
